# Alteration of immunophenotype of human macrophages and monocytes after exposure to cigarette smoke

**DOI:** 10.1038/s41598-020-68753-1

**Published:** 2020-07-30

**Authors:** Camila Oliveira da Silva, Thomas Gicquel, Yoann Daniel, Thiago Bártholo, Elise Vène, Pascal Loyer, Luís Cristóvão Pôrto, Vincent Lagente, Tatiana Victoni

**Affiliations:** 1 Laboratory of Histocompatibility and Cryopresevation, Rio de Janeiro State University, Rio de Janeiro, Brazil; 2 NuMeCan Institute (Nutrition, Metabolism and Cancer), INSERM, INRAE, CHU Rennes, Hôpital Pontchaillou, Univ Rennes, 35033 Rennes Cedex, France; 3 Forensic and Toxicology Laboratory, Pontchaillou University Hospital, Rennes, France; 4 Department of Pulmonology, Rio de Janeiro State University, Rio de Janeiro, Brazil; 5 University of Lyon, VetAgro Sup, APCSe, Marcy l’Étoile, France

**Keywords:** Cell biology, Immunology, Molecular biology, Molecular medicine

## Abstract

Cigarette smoke exposure (CS) is the main risk factor for chronic obstructive pulmonary disease (COPD). Macrophages have an important role in COPD because they release pro-inflammatory and anti-inflammatory cytokines. The present study’s we investigate the functional changes in macrophages and monocytes exposed to cigarette smoke extract (CSE). Herein, using human monocyte-derived macrophages (MDMs) from healthy donors and we found that CSE was not associated with significant changes in the production of pro inflammatory cytokines by MDMs. In contrast, exposure to CSE suppressed the production of IL-6 and Gro-a/CXCL1 by LPS-stimulated-MDMs, but had an additive effect on the release of IL-8/CXCL8 and MCP1/CCL2. However, CSE exposure was associated with greater production, TARC/CCL-17 and CCL22/MDC. Moreover, MDMs displayed a lower uptake capacity after CSE exposure. We identify, for what is to our knowledge the first time that monocytes from patients with COPD produced less IL-8/CXCL8 and Gro-α/CXCL1 after LPS stimulation and produced higher levels of TARC/CCL17 and MDC/CCL-22 after IL-4 stimulation. Our present results highlighted a skewed immune response, with an imbalance in M1 vs. M2 cytokine production. In conclusion, exposure to CS has contrasting, multifaceted effects on macrophages and monocytes. Our data may provide a better understanding of the mechanisms underlying COPD.

## Introduction

Chronic obstructive pulmonary disease (COPD) is now the world’s third-leading cause of mortality^1^. The disease is characterized by persistent respiratory symptoms, airflow limitation, and airway and/or alveolar abnormalities associated with an inflammatory response^2^. Exposure to cigarette smoke (CS) is the main risk factor for the development of COPD^3^. It has been shown that exposure to CS can induce an influx of inflammatory cells, which then aggravates the inflammatory processes observed in COPD^4^. In contrast, CS also suppresses local innate host defenses in the airway^5^. A number of recent studies have focused on how CS distorts the immune response, and many have highlighted the role of macrophages and monocytes as effector cells^6–8^.

Generally, macrophages consist of two polarization states. Activated M1 macrophages (also referred to as “classically activated” macrophages) produce pro-inflammatory cytokines such as tumor necrosis factor (TNF) α, IL-6 and IL-12, whereas activated M2 macrophages (also referred to as “alternatively activated” macrophages) produce anti-inflammatory molecules such as IL-10 and transforming growth factor β^9–11^. Differential cytokine production is a key feature of polarized macrophages: IL-8/CXCL8, IL-6, TNF-α, and the macrophage-derived chemokine CXCL1 are hallmarks of the M1 phenotype, while the macrophage-derived chemokine CCL22, thymus- and activation-regulated chemokine CCL-17, and IL-10 are markers of the M2 phenotype^10,11^.

Renewal of macrophages depending blood monocytes that are recruited to the lung after injury, these new migrant monocytes can mature (i.e. polarize) into distinct macrophage subpopulations with divergent functional activities. It has been suggested that the microenvironment could induce polarization states. Another possibility is that distinct populations of blood monocytes are attracted to inflamed tissues, where they give rise to macrophage populations^12^. Indeed, the existence of monocyte subsets with distinct roles in homeostasis and inflammation is suggestive of functional specialization. Classical CD14^++^/CD16^−^ monocytes appear to be dedicated to phagocytosis, the production of reactive oxygen species (ROS), and the secretion of pro-inflammatory cytokines^13^, whereas nonclassical CD14^+^/CD16^++^ cells are more like resident tissue macrophages^14^. In different chronic diseases (such as atherosclerosis, rheumatoid arthritis and COPD); the circulating monocytes have different phenotypes^14–17^.

Macrophages polarization is accompanied by changes of phagocytose and euferocytoses capacity. It has been reported that CS can perturb phagocytosis and efferocytosis in macrophages. Hence, impaired phagocytosis and efferocytosis of apoptotic cells may contribute to exacerbations and the progression of COPD^18–20^.

The role of macrophage polarization in respiratory diseases has been extensively discussed^21–23^. Polarized changes are less apparent in COPD, although the dysregulation of M1 and M2 polarization patterns has been described—the upregulation and downregulation of both M2-related and M1-related genes by macrophages, and the unexpected absence of inflammatory signatures in alveolar macrophages obtained from patients with COPD who smoke (relative to non-COPD smokers)^23^. However, levels of inflammation appear to abnormally high in patients with COPD-particularly during acute exacerbations^24^.

Much of our knowledge about the effect of CS on macrophage polarization has been generated in experiments on animal models and cell lines^22,25^. Although these studies may provide mechanistic insights, their actual relevance to human disease is largely unknown. Some data from human macrophages has been described, but most were based on gene expression^23^. Little is known about macrophage function (especially the ability to release cytokines) in a COPD setting. Moreover, the impact of the monocyte’s phenotype on cytokine release has not been determined.

The objective of the present study was to explore the functional effects of CS exposure on macrophages and monocytes. We examined M1 and M2 cytokine production and uptake in human monocyte-derived macrophages (MDMs) from healthy donors after exposure to CS extract (CSE). Moreover, we explored the effect of concomitant exposure to CSE and M1 and M2 stimuli. Lastly, we determined whether M1 and M2 cytokine production is altered in monocytes taken from patients with COPD.

## Results

### The effect of exposure to CSE on the production of Gro-α/CXCL1, IL-6, IL-8/CXCL8, MCP-1/CCL2, TNF-α, IL-10, MDC/CCL22, TARC/CCL17 and PARC/CCL18 by MDMs

**Figure 1:**
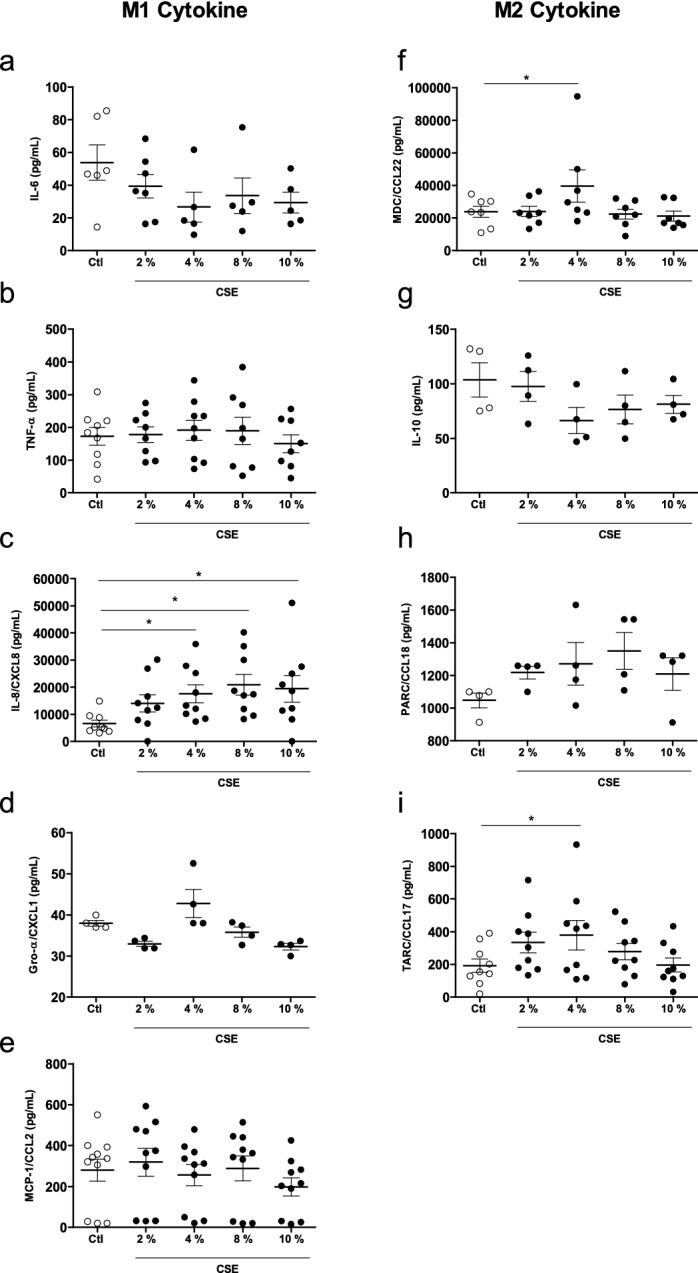
Effects of CSE on cytokine production by MDMs. The MDMs were incubated with medium alone (control) or with different concentration of CSE (2–10%) for 24 h. The culture supernatants were collected, and the concentrations of IL-6, TNF-α, IL-8/CXCL8, Gro-α/CXCL-1, MCP-1/CCL-2, MDC/CCL22, IL-10, PARC/CCL8, and TARC/CCL17 were measured using an ELISA. The data were assessed in an ANOVA, followed by a Bonferroni post-test. The data correspond to the mean ± SEM of 4–10 donors. *p < 0.05, compared with the control.

The incubation of MDMs with different concentrations of CSE (2%, 4%, 8% and 10%) did not appear to affect the production of M1 cytokines such as IL-6, TNF-α, CXCL1/Gro-α and MCP-1/CCL2 (Fig. 1a,b,d,e). In contrast, the cells’ production of IL-8/CXCL8 increased in a concentration-dependent manner (Fig. 1c).

With regard to M2 cytokines, a 24-h exposure to CSE did not accentuate the release of IL-10 or PARC/CCL18, when compared with a control (Fig. 1g,h). However, exposure to 4% CSE was associated with greater MDC/CCL22 and TARC/CCL17 release (Fig. 1f,i). Moreover, cell viability was not affected by CSE concentrations of 2%, 4% and 8%. However, number of viable cells fell at a concentration of 10% (data not shown).

Given that 4% CSE induced the secretion of most of the cytokines studied and did not have a toxic effect, this concentration was selected for the subsequent experiments.

### Effects of the CSE-LPS combination on cytokine gene expression and protein production by MDMs

**Figure 2:**
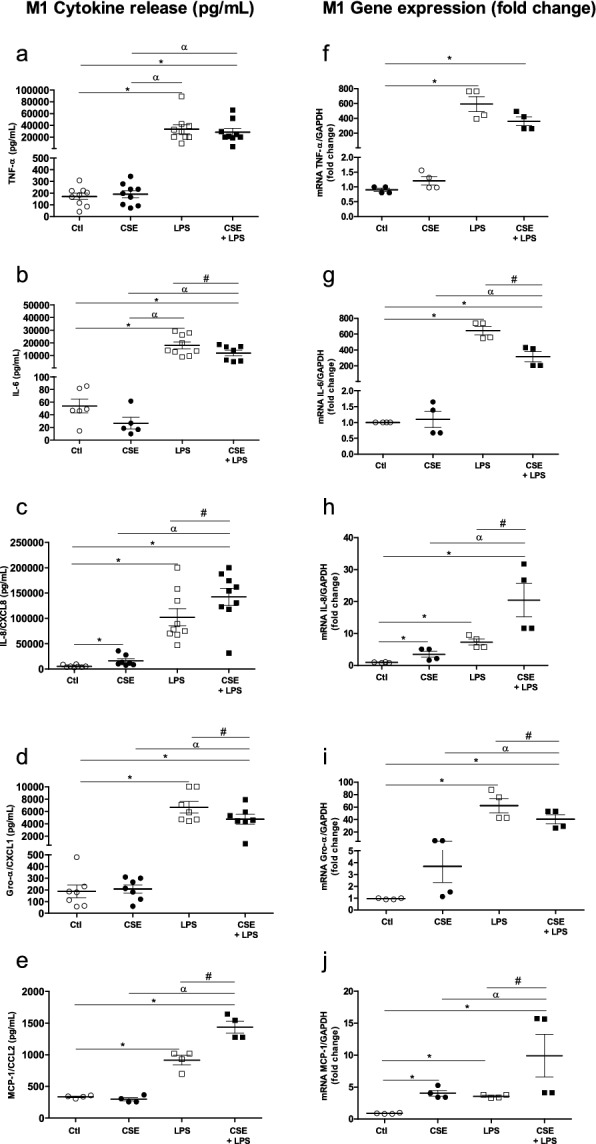
Effects of a combination of CSE and LPS on M1 cytokine production and expression by MDMs. The MDMs were incubated with medium alone (control), 4% CSE, 0.1 µg/mL LPS or 4% CSE + 0.1 µg/mL LPS for 2 h or 24 h. The culture supernatants were collected, and the concentrations of TNF-α, IL6, IL-8/CXCL8, Gro-α/CXCL1 and MCP-1/CCL-2 were measured using an ELISA (**a**–**e**). MDMs were stimulated for 2 h, and mRNA expression was determined using RT-PCR (**f**–**j**). The results were normalized against expression of the *GAPDH* gene. The data were assessed in an ANOVA, followed by a Bonferroni post-test. The data correspond to the mean ± SEM of 4–9 donors. *p < 0.05 compared with the control; ^α^p < 0.05 compared with 4% CSE; ^#^p < 0.05, compared with 0.1 µg/mL LPS.

Exposure to 4% CSE did not appear to affect M1 cytokine release, with the exception of IL-8/CXCL8. Hence, we sought to establish the impact of exposure to CSE on the immune response by investigating cytokine release by LPS-stimulated MDMs. As expected, LPS at a concentration of 0.1 µg/mL induced the expression and production of all the M1 cytokines, relative to control experiments (Fig. 2). Interestingly, however, CSE inhibited IL-6 and Gro-a/CXCL1 expression and production in LPS-stimulated MDMs (Fig. 2b,d,g,i) but did not affect TNF-α expression and production (Fig. 2a,f). In contrast, CSE had an additive effect on IL-8/CXCL8 and MCP-1/CCL2 release and mRNA expression in LPS-stimulated MDMs (Fig. 2c,h,e,j).

**Figure 3:**
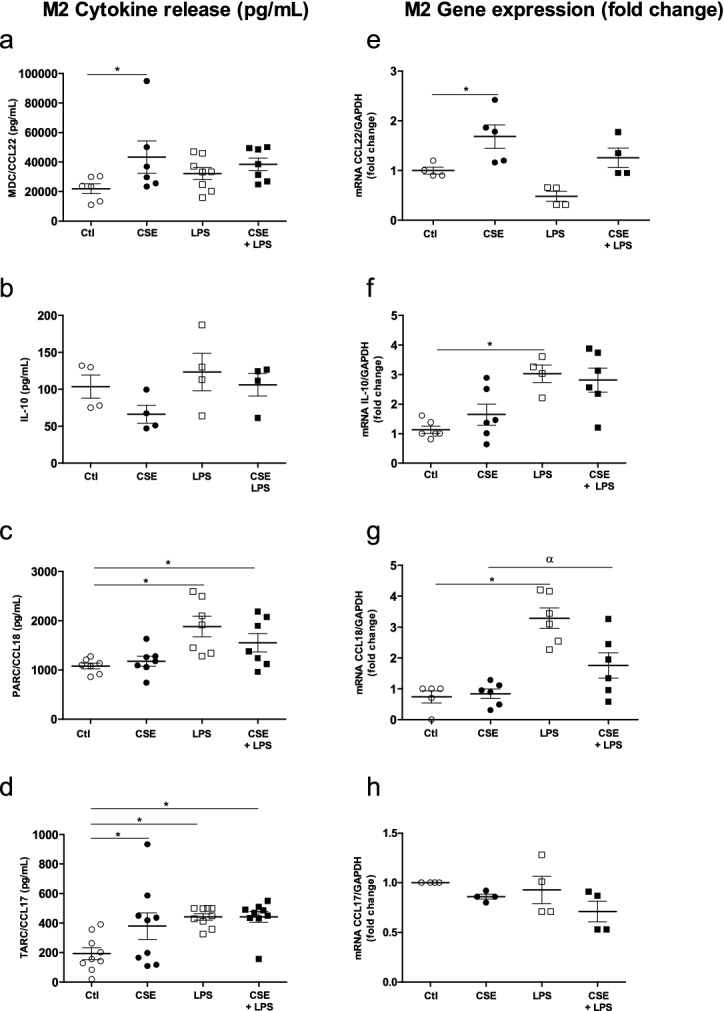
Effects of a combination of CSE and LPS on M2 cytokine production and expression by MDMs. The MDMs were incubated with medium alone (control), 4% CSE, 0.1 µg/mL LPS, or 4% CSE + LPS for 24 h. The culture supernatants were collected, and the concentrations of MDC/CCL22, IL-10, PARC/CCL8 and TARC/CCL17 were measured using an ELISA (**a**–**d**). MDMs were stimulated for 2 h, and mRNA expression was then determined using RT-PCR (**e**–**h**). The results were normalized against expression of the *GAPDH* gene. The data were assessed in an ANOVA, followed by a Bonferroni post-test. The data correspond to the mean ± SEM of 4 to 9 donors. *p < 0.05, compared with the control; ^α^p < 0.05, compared with 4% CSE; ^#^p < 0.05, compared with 0.1 µg/mL LPS.

Next, we investigated the effect of exposure to CSE on M2 cytokine production by LPS-stimulated MDMs. We found that the exposure to 4% CSE induced the release and mRNA expression of MDC/CCL22 (Fig. 3a,e) but not release and mRNA expression of IL-10 and PARC (Fig. 3b,c,f,g). Moreover, only TARC/CCL17 release was increase by CSE, and no change in the expression of TARC/CCL17 was observed (Fig. 3d,h). Furthermore, 0.1 µg/mL LPS induced mRNA expression of IL-10 and PARC/CCL18 (Fig. 3f,g), and the release of PARC/CCL18 and TARC/CCL17 (Fig. 3c,d) when compared with controls. Lastly, incubation with LPS did not change MDC/CCL22 release and expression or IL-10 release (Fig. 3a,e,b).

The exposure of LPS-stimulated MDMs to 4% CSE for 24 h did not affect the release and expression of MDC/CCL22, IL-10 and TARC/CCL17, when compared with CSE or LPS alone (Fig. 3a,b,d,e,f,h). In contrast, LPS-stimulated MDMs exposed to 4% CSE expressed low levels of PARC/CCL18, relative to MDMs exposed to LPS alone (Fig. 3g).

### Effects of combining CSE with an M2 stimulus (IL-4) on cytokine gene expression and protein production by MDMs

Given that exposure to CSE appeared to have an immunosuppressive effect on some M1 cytokines but also induced M2 cytokines (such as MDC/CCL22 and TARC/CCL17), we next investigated the effects of exposure to CSE on IL-4 stimulated MDMs.

**Figure 4:**
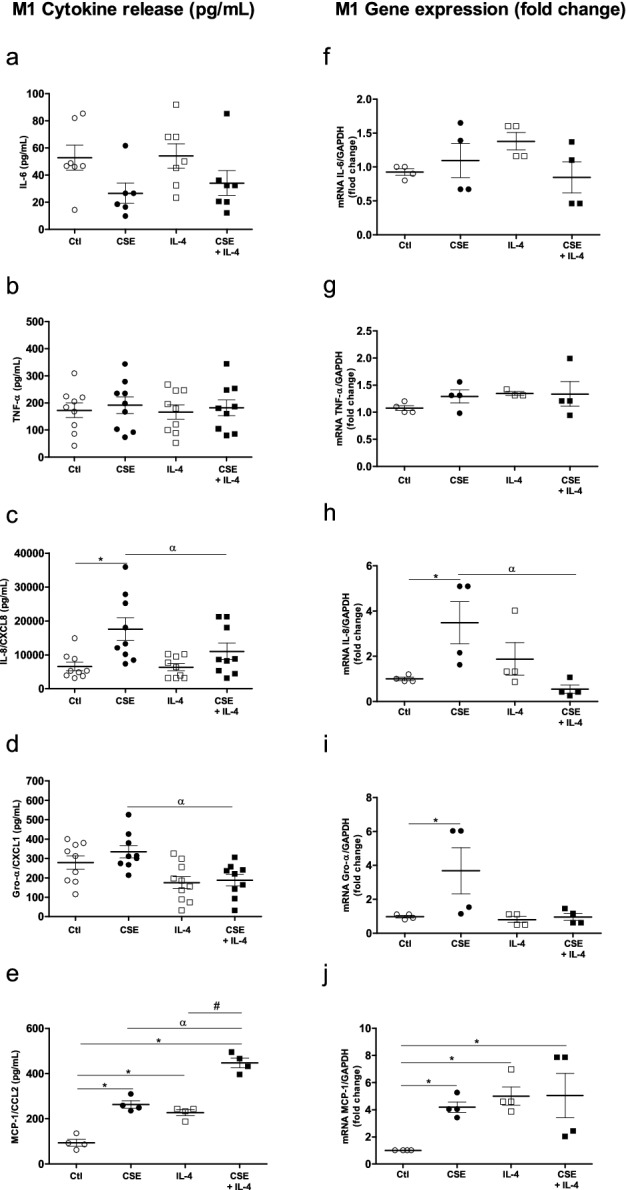
Effects of a combination of CSE and IL-4 on M1 cytokine production and expression by MDMs. The MDMs were incubated with medium alone (control), 4% CSE, 10 ng/mL IL-4 or CSE + IL-4 for 24 h. The culture supernatants were collected, and the concentrations of IL-6, TNF-α, IL-8/CXCL8, Gro-α/CXCL1 and MCP-1/CCL-2 were measured using an ELISA (**a**–**e**). mRNA expression was then determined using RT-PCR (**f**–**j**). The results were normalized against expression of the *GAPDH* gene. The data were assessed in an ANOVA, followed by a Bonferroni post-test. The data correspond to the mean ± SEM of 9 donors. *p < 0.05 compared with the control; ^α^p < 0.05, compared with 4% CSE; ^#^p < 0.05, compared with IL-4.

The exposure of MDMs in the presence of 10 ng/mL IL-4 did not induce the expression and release of M1 cytokines, with the exception of MCP-1/CCL2 (Fig. 4e,j). Furthermore, CSE exposure had no effect on the release and expression of IL-6 and TNF-α by IL-4-stimulated MDMs when compared with controls or CSE exposure alone (Fig. 4a,b,f,g).

In contrast, exposure to CSE and IL-4 produced relative decreases in the release and expression of IL-8/CXCL8 and Gro-α/CXCL1 by MDMs, when compared with CSE alone (Fig. 4c,d,h,i). Furthermore, exposure to 4% CSE had an additive effect on MCP-1/CCL2 release by IL-4-stimulated MDMs (Fig. 4e).

**Figure 5:**
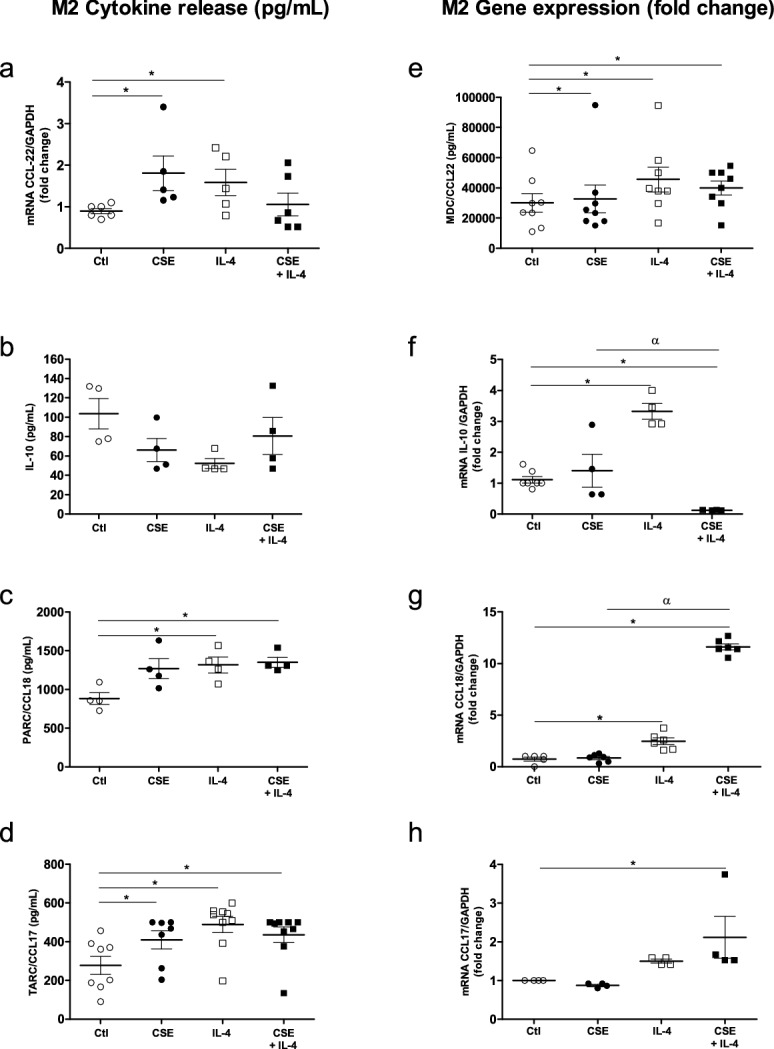
Effects of a combination of CSE and IL-4 on M2 cytokine production and expression by MDMs. The MDMs were incubated with medium alone (control), 4% CSE, 10 ng/mL IL-4 or 4% CSE + IL-4 10 µg/mL for 24 h. The culture supernatants were collected and the concentrations of MDC/CCL22, TARC/CCL17, IL-10, and PARC/CCL8 were measured using an ELISA (**a**–**d**). mRNA expression was then determined using RT-PCR (**e**–**i**). The results were normalized against expression of the *GAPDH* gene. The data were assessed in an ANOVA, followed by a Bonferroni post-test. The data correspond to the mean ± SEM of 9 donors. *p < 0.05 compared with the control; ^α^p < 0.05, compared with 4% CSE; ^#^p < 0.05 compared with 10 ng/mL IL-4.

With regard to M2 cytokines, the incubation of MDMs with 10 ng/mL IL-4 for 24 h enhanced the release of MDC/CCL22, PARC/CCL18 and TARC/CCL17 (Fig. 5a,c,d) and the expression of MDC/CCL22, IL-10 and PARC/CCL18, when compared with controls (Fig. 5e,f,g). However, the IL-4 stimulus had no effect on IL-10 release or TARC/CCL17 expression (Fig. 5b,h). Moreover, exposure to CSE had no effect on cytokine release by IL-4-stimulated MDMs. We only observed an additive effect of CSE exposure on TARC/CCL17and PARC/CCL18 gene expression in IL-4-stimuled MDMs (Fig. 5g,h) when compared with IL-4 alone or controls, respectively. Moreover, we observed a decrease in the expression of IL-10 after CSE exposure of IL-4 -stimulated MDMs (Fig. 5f).

### Exposure to CSE alters macrophages’ uptake capacity

**Figure 6:**
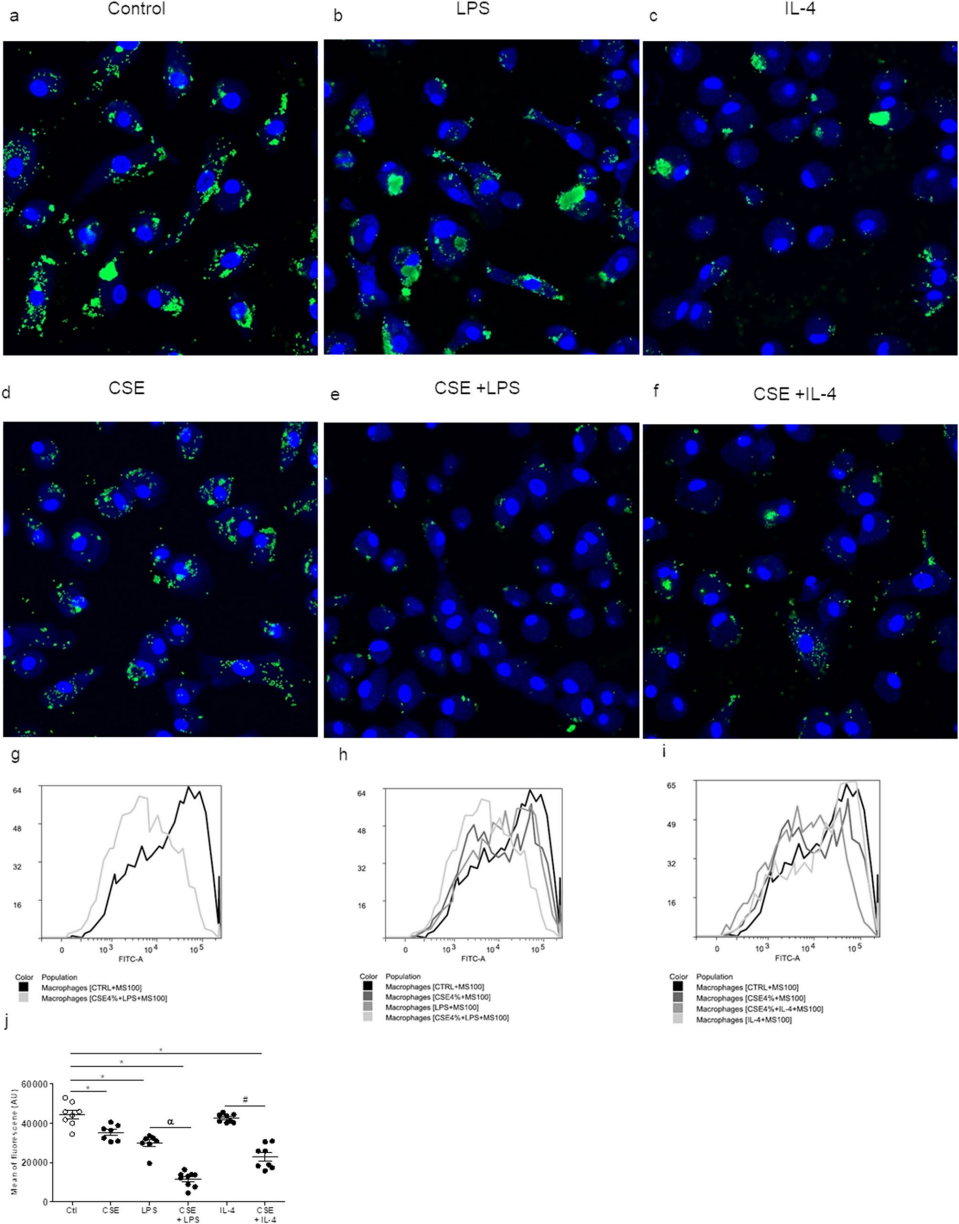
Effects of CSE, CSE + LPS and CSE + IL-4 on microsphere uptake by MDMs. After 24 h of exposure to CSE, the culture medium was renewed with medium containing fluorescent microspheres (size: 100 nm) and incubated overnight. After incubation, the culture medium was discarded, and the cell monolayers was fixed with paraformaldehyde prior to observation under the confocal microscope (**a**–**f**). The fluorescence emitted by microspheres (on channel FL1-H) inside cells was analyzed using CellQuest cytometry software (**g**–**i**). The intrinsic FL1-H fluorescence of MDMs was measured in the absence of microspheres. The data correspond to the mean ± SEM of representative experiment of one the 4 donors. *p < 0.05, compared with the control; ^α^p < 0.05, compared with LPS; ^#^p < 0.05, compared with CSE + IL-4.

After studying the CSE-stimulated MDMs’ ability to release cytokines, we focused on the cell’s ability to capture microspheres. The incubation of MDMs with CSE and LPS for 18 h decreased the uptake capacity, when compared with a control (Fig. 6a,b,d,e,g,h,j). The presence of IL-4 alone did not affect uptake cell capacity (Fig. 6c,f,i,h). Moreover, microsphere uptake was reduced further when cells were exposed to both CSE and LPS or to both CSE and IL-4 (Fig. 6e,f,j).

### Profile of monocytes from patients with COPD, and the production of M1 and M2 cytokines

**Figure 7:**
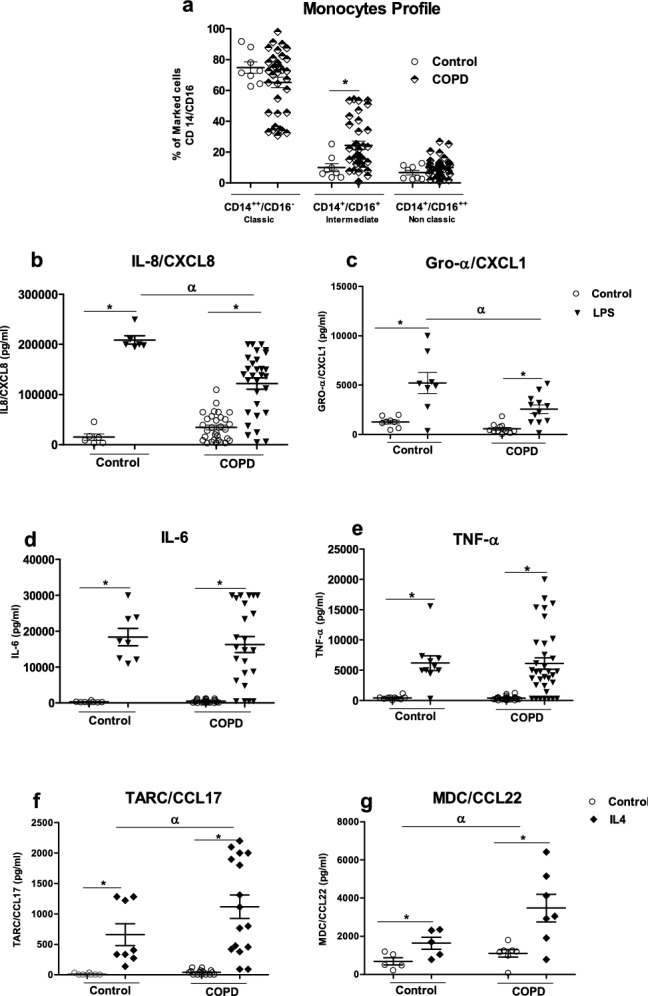
Subpopulations of peripheral blood monocytes in healthy subjects and patients with COPD and the cells’ ability to release cytokines after M1 or M2 activation. Subpopulations of monocytes were determined by flow cytometry. The monocytes were stained for anti-CD14, anti-CD16 and anti-HLADR. The data are presented as the percentages of total classical (CD14^++^/CD16^−^), intermediate (CD14^+^/CD16^+^) and non-classical (CD14^+^/CD16^++^) monocytes for each group (**a**). The total monocyte fraction was incubated with medium alone (control), 0.1 µg/mL LPS or 10 ng/mL IL-4 for 24 h. The culture supernatants were collected, and the concentrations of IL-8/CXCL8, IL-6, Gro-α/CXCL1, TNF-α, MDC/CCL22, and TARC/CCL17 and were measured using an ELISA (**b**–**g**). The data were assessed in an ANOVA, followed by a Bonferroni post-test. The data correspond to the mean ± SEM of 8 to 37 donors. *p < 0.05, compared with the control; ^α^p < 0.05, compared with LPS or IL-4.

In COPD, macrophages accumulate in the airways; peripheral monocytes may then have to replenish lung macrophages^26^. We therefore profiled of blood monocytes obtained from patients with COPD with regard to the expression of CD14 and CD16^13^. The samples from patients with COPD had a higher intermediate monocyte (CD14^+^/CD16^+^) count than samples from healthy donors. However, there were no intergroup differences between in the counts of classical monocytes (CD14^++^/CD16^−^) and nonclassical monocytes (CD14^+^/CD16^++^) (Fig. 7a).

Next, we explored the monocytes’ ability to release cytokines. Again, a COPD background did not appear to affect cytokine release. There was no difference between COPD monocytes and those from healthy donors with regard to the release of IL-8/CXCL8, IL-6, Gro-α/CXCL1, TNF-α, MDC/CCL22, and TARC/CCL17 (Fig. 7b–g). However, monocytes from patients with COPD incubated with 0.1 µg/mL LPS for 24 h produced less IL-8/CXCL8 and Gro-α/CXCL1 than monocytes from healthy donors did (Fig. 7b,c). In contrast, COPD monocytes stimulated with 10 ng/mL IL-4 for 24 h produced more MDC/CCL22 and TARC/CCL17 than healthy donor monocytes did (Fig. 7f,g). Lastly, there was no marked difference in IL-6 and TNF-α release after exposure to LPS (Fig. 7d,e).

## Discussion

It has long been held that the progression of COPD is essentially due to “inflammation”. This is true but only in part; there is now evidence to show that the inflammatory hypothesis is over-simplistic at the very least. In the present study, we found that the activation of macrophages and monocytes by CS was mechanistically complex and involved both pro-inflammatory and immunosuppressive processes. The cytokine release profiles documented here might provide a better understanding of COPD and the contribution of monocytes and macrophages to tissue remodeling and disease progression in an inflammatory context.

In the present study, we primarily studied cytokine expression and release by MDMs after CSE exposure and by monocytes from patients with COPD. We found that CSE exposure or smoking did not appear to affect M1 or M2 cytokine production by MDMs or monocytes. Furthermore, exposure to CSE changed the ability of the LPS- or IL-4 stimulated cells to produce cytokines. The present study is the first to show that exposure to CS can also effect monocytes’ ability to release both pro-inflammatory and immunoregulatory cytokines. Lastly, we showed that exposure to CSE alone or CSE combined with an M1 or M2 stimulus impairs MDM’s ability to capture microspheres.

Our observations of cytokine release corroborate the gene expression results reported by Shaykhiev et al. The latter researchers found that alveolar macrophages from healthy smokers exhibited a polarization pattern with the downregulation of M1-related inflammatory genes and the upregulation of genes associated with M2-polarization programs of relevance in tissue and immune regulation^23^. However, some cytokines are exceptions, it has been reported that exposure to CS is associated with the elevated release of IL-8/CXCL8 by MDMs and alveolar macrophages^27,28^. The literature data indicate that the stimulation of smoke’ alveolar macrophages with TLR2 and TLR4 agonists leads to elevated production of IL-8/CXCL8 and RANTES/CCL5 and downregulation of IL-6 and TNF-α; our present results corroborate those findings, although we did not observe a difference in the level of TNF-α after exposure to CSE alone or to CSE and LPS^29^.

High levels of CCL2/MCP1 and CXCL1/Gro-α are typically found in the airways of patients with COPD^30^. We found that LPS-stimulated MDMs released high levels of the chemokines MCP1/CCl2 but not Gro-α/ CXCL1. This discrepancy might be due to the chemokines’ respective roles. MCP-1/CCL2 is a specific CXC chemokine; it has a chemotactic effect on macrophages and basophils, and can increase the number of migrating monocytes in the context of COPD^31^. MCP1/CCL2 is also known to have effects on tissue remodeling. Furthermore, recent and ongoing research indicates that MCP-1/CCL2 has in other aspects of the inflammatory process, such as fibrosis and tissue remodeling^32^. Gro-α/CXCL (a specific CC chemokine) has strong neutrophil chemoattractant activity in acute inflammation^32^. An increase in MCP1/CCL2 levels and a decrease in Gro-α/ CXCL levels is consistent with the immunosuppressive effects of CS. However, it is not clear why the CSE’s downregulatory on pro-inflammatory cytokines applied to CXCL1/Gro-α but not IL-8/CXCL8, even when both chemokines are responsible for recruiting neutrophils. It may be that the chemotactic mechanisms are different. It has also been shown that in the context of COPD, IL-8/CXCL8 induction by CSE has a greater role in neutrophilic lung inflammation than Gro-α/CXCL1^33^.

After observing that co-stimulation with CSE and LPS alters cytokine production, we then investigated the effect of CSE on cytokine release by IL-4-stimulated MDMs. With regard to IL-8/CXCL8 production, co-stimulation with CSE and IL-4 had the opposite effect to co-stimulation with LPS and CSE. This difference might be explained by the mechanism of downregulation. It has been shown that when cells are exposed to IL-4 and IL-13, increased receptor expression and coupling render the cells extremely sensitive to IL-8/CXCL8 and related CXC chemokines. However, the combination of CSE and IL-4 had an additive effect on MCP1/CCL2 release. Given the dual role of this CC chemokine mentioned above (tissue remodeling and macrophage recruitment), this result is not unexpected.

We also investigated the effect of CSE and IL-4 on the release of M2 cytokines. At a dilution of 4% (but not at other dilutions), CSE induced MDC/CCL22 and TARC/CCL17 release. This result can be explained by the toxicity of high concentrations of CSE (data not shown). Ours results are in line with the reports by Ying et al.^34^ and Eapen et al.^21^, who observed elevated TARC/CCL17 and MDC/CCL22 levels in the BAL of smokers and ex-smokers with COPD. Similarly, lung expression of TARC/CCL17 and MDC/CCL22 was selectively elevated in an animal model of chronic exposure to CS^35^. Nevertheless, CSE did not have a direct effect on IL-10 and PARC; accordingly, Chen et al*.* showed that the expression of IL-10 in alveolar macrophages stimulated with TLR2 and TLR4 agonists was not affected by smoking^29^.

We observed disparities between the cytokine release and gene expression results for PARC and IL-10; we have three possible explanations for this. Firstly, the time exposure was short; longer exposure might have been necessary for observing the additive upregulatory effects of CSE. Secondly, the increase in gene expression might not have been large enough to enhance cytokine release. Thirdly, post-transcriptional mechanisms might have modified the cytokine release of this.

Like Cornwell et al.^15^, we found that the proportion of intermediate monocytes was higher in patients with COPD than in controls. However, we also found that LPS-stimulated monocytes from patients with COPD released lower levels of IL-8/CXCL8 and Gro-α/CXCL1 when compared with healthy donor monocytes. However, IL-4-stimulated monocytes from patients with COPD released higher levels of TARC/CCL17 and MDC/CCL2 than cells from healthy donors. To the best of our knowledge, the present study is the first to have evidenced functional differences in cytokine release by monocytes from patients with COPD.

These results are in line with other earlier report in which mice exposed to CS had elevated numbers of non-classical monocytes in the blood and bone marrow and M2 macrophages in the lung^36^. We hypothesize that intermediate monocytes (present in abnormally high numbers in patients with COPD) are predisposed to differentiate into M2 macrophages in the lung. However, further studies are needed to determine whether intermediate monocytes from blood become M2 macrophages in the lungs.

The available evidence suggests that exposure to CSE not only modifies cytokine release but also impairs phagocytosis and efferocytosis^5,6^. We found that exposure did impair uptake by MDMs (including those stimulated by LPS or IL-4) but do not have a mechanistic explanation for this loss of function. It was recently reported that CS attenuates phagocytosis by macrophages via the downregulation of milk fat globule-EGF factor 8 (MFG-E8) expression; a protein that facilitates efferocytosis^19^. We suggest also that the excessive oxidative stress produced by CS harms macrophage function and thus impairs the phagocytosis of bacteria and efferocytosis of apoptotic cells. Hence, impaired phagocytosis and efferocytosis of apoptotic cells may contribute to exacerbations and the progression of COPD.

In conclusion, our present results highlighted a skewed immune response, with an imbalance in M1 vs. M2 cytokine production and worsened uptake. However, exposure to CSE has contrasting, multifaceted effects on macrophages and monocytes, and does not fit with the conventional M1/M2 dichotomy. Lastly, the monocytes’ profile might contribute to this disturbance of cytokine release, and might have a role in the physiopathology of COPD. Our data may provide a better understanding of the complex mechanisms underlying COPD and thus facilitate the development of novel treatments for this condition.

## Materials and methods

### Subject selection

All methods were carried out in accordance with the relevant guidelines and all subjects gave their written informed consent to participate in the study. Healthy blood donors (control participants) with negative serologic tests for syphilis, hepatitis B and C, and HIV were recruited by the Hemotherapy Unit at Pedro Ernesto University Hospital (Rio de Janeiro, Brazil). Patients with COPD were recruited by the Department of Pulmonology and Urology at Pedro Ernesto University Hospital. Patients with COPD had to have a forced expiratory volume in 1 s (FEV1) of between 30 and 80% predicted, were aged 50 or over, and had a spirometry-proven diagnosis of COPD and a smoking burden of more than 20 pack-years. An obstructive pulmonary disorder corresponds to an FEV1 (forced first–second expiratory volume)/forced vital capacity (FVC) ratio of less than 0.7 after the use of salbutamol 400 µg. The control group comprised individuals aged 50 or over, with normal spirometry and chest X-ray results and no airflow obstructions. The demographic characteristics of the study population are summarized in Table 1.

**Table 1:** The demographic characteristics of the study population.

Severity GOLD GRADE	Age (years)	Gender (M/F)	FEV_1_ (% Pred)	Current smoke (Y/N)
Normal	(50–70)	(5/3)	≥ 80	(0/8)
COPD-1	70 (56–85)	(11/5)	≥ 80	(2/14)
COPD-2	66 (53–87)	(11/9)	60.7 (51–77)	(7/13)
COPD-3	66 (54–83)	(6/4)	40.1 (31–49)	(3/7)
COPD-4	65 (56–79)	(5/0)	20.8 (23–29)	(0/5)

Patients with COPD were recruited by the Department of Pulmonology and Urology at Pedro Ernesto University Hospital. Patients with COPD had to have a forced expiratory volume in 1 s (FEV1) of between 30 and 80% predicted, were aged 50 or over, and had a spirometry-proven diagnosis of COPD and a smoking burden of more than 20 pack-years. An obstructive pulmonary disorder corresponds to an FEV1 (forced first–second expiratory volume)/forced vital capacity (FVC) ratio of less than 0.7 after the use of salbutamol 400 µg. The control group comprised individuals aged 50 or over, with normal spirometry and chest X-ray results and no airflow obstructions.

### Cell culture

Primary human MDMs were obtained by differentiating peripheral blood mononuclear cells (PBMCs) in buffy coat donations (from the French Blood Agency, *Etablissement Français du Sang*, Rennes, France, and the Hemotherapy Unit at Pedro Ernesto University Hospital ), as described previously^37^. All experiments complied with the French legislation on blood transfusion safety (French Government Act 93-5 dated January 4th, 1993), and were approved by the French Blood Agency and the above-mentioned institutional review board Piquet Carneiro Ethics Committee (Rio de Janeiro, Brazil); reference: 44684515.3.0000.5259), and all subjects gave their written informed consent to participate in the study.

Briefly, PBMCs were harvested from the human buffy coat using Ficoll centrifugation (GE Healthcare Life Sciences, Little Chalfont, UK). Next, the monocytes were enriched using a human CD14 microbead separation kit (Miltenyi Biotec, Bergisch Gladbach, Germany), and resuspended in RPMI 1,640-Glutamax medium (Sigma-Aldrich, St. Louis, MO, USA) supplemented with 1% glutamine, 0.1% penicillin, 0.5% streptomycin (Invitrogen Eugene, OR, USA) and 10% fetal bovine serum, and placed in 24-well plates at a density of 0.5 × 10^6^ cells/well. To obtain MDMs, the monocytes were incubated with 50 ng/mL of granulocyte-monocyte colony stimulating factor (GM-CSF) (R&D Systems, Lille, France) for 7 days. Subsequently, the culture medium was removed and the macrophages were deprived of GM-CSF for 24 h before stimulation experiments.

### Preparation of CSE

The CSE was prepared as previously described^25^. In brief, the smoke from two cigarettes (Marlboro Red, containing 13 mg of tar, 1 mg of nicotine, and 10 mg of carbon monoxide on average) was aspirated with the aid of a peristaltic pump and placed in contact with 20 mL of culture medium. The medium was then filtered through gauze to remove larger particles and then sterilized through a 0.2 μM filter. The solution was standardized to pH 7 and an absorbance of 1.5–2.0 at a wavelength of 326 nm. This 100% CSE stock solution was diluted to 2%, 4%, 8% and 10% immediately prior to experiments.

### Treatments

Monocyte-derived macrophages from healthy donors were exposed to different dilutions of CSE (2%, 4%, 8% and 10%), LPS (0.1 μg/mL; from E. coli 055: B5, Sigma-Aldrich), IL-4 (10 ng/mL; R&D Systems) alone or in combinations (4% CSE + LPS 0.1 μg/mL or 4% CSE + 10 ng/mL IL-4) for 2 h or 24 h. Monocytes from patients with COPD were exposed to LPS 0.1 μg/mL or 10 ng/mL IL-4 for 24 h. The supernatant was collected, and the cells were left on the plate, stored at − 80 °C, and thawed immediately prior to cytokine production and gene expression assays.

### The cell viability assay

The cytotoxicity of CSE for MDMs was assessed in a tetrazolium salt (3-(4,5-dimethylthiazol-2-yl)-2,5-diphenyltetrazolium bromide, MTT) cell viability assay. After exposure to CSE, the culture medium was aspirated and the cells were incubated with MTT (Sigma-Aldrich) at a final concentration of 0.5 mg/mL for 1 h at 37 °C and 5% CO_2_. The product of the formazan reaction was extracted into DMSO (Sigma-Aldrich), the optical density was measured spectrophotometrically at 540 nm with DMSO as the blank. Viability was expressed as a percentage of the value obtained with untreated cells (i.e. control = 100%).

### Cytokine and chemokine assays

The concentrations of IL-6, TNF-α (25–900-K21, Peprotech, Rocky Hill, NJ, USA), Growth-regulated oncogene (Gro)-α/CXCL1, IL-8/CXCL8, IL-10, monocyte chemoattractant protein 1 (MCP)1/CCL2, Macrophage-derived chemokine (MDC)/CCL22, pulmonary and activation-regulated chemokine (PARC)/CCL18, and thymus and activation regulated chemokine (TARC)/CCL17 (all from R&D Systems) in the culture supernatants were measured using commercial ELISA kits, according to the manufacturer's instructions. The supernatant was diluted to immediately prior to experiments as described in Table 2

**Table 2:** Dilution used for specific cytokines for each conditions.

Cytokine	MDM						Monocytes	
	Control	CSE 4%	LPS	LPS + CS4%	IL-4	IL-4 + CS4%	Control	LPS
	Dilution						Dilution	
IL-6	1:1	1:1	1:200	1:200	1:1	1:1	1:2	1:50
TNF-α	1:1	1:1	1:100	1:100	1:1	1:1	1:1	1:10
IL-8/CXCCL8	1:10 or 1:100	1:100	1:100 or 1:200	1:100	1:100	1:100	1:100	1:100
Gro-α/CXCL1	1:1	1:1	1:100	1:100	1:1	1:1	1:2	1:10
MCP1/CCL2	1:4	1:4	1:20	1:20	1:4	1:4	-	-
MDC/CCL22	1:100	1:100	1:100 or 1:200	1:100 or 1:200	1:100 or 1:200	1:100 or 1:200	1:100	1:100
IL-10	1:1	1:1	1:1	1:1	1:1	1:1	-	-
PARC/CCL18	1:10	1:10	01:10	01:10	01:10	1:10	-	-
TARC/CCL17	1:1	1:1	1:1	1:1	1:1	1:1	1:2	1:2

The supernatant was diluted to immediately prior to experiments.

### Real-time quantitative PCR (RT-qPCR) analysis

Total RNA was isolated from the cells using a commercially available kit (NucleoSpin RNAII from Macherey–Nagel, Dueren, Germany), as previously described^25^. The quantity and purity of the RNA were measured with a Nanodrop ND-1000 spectrophotometer (Nyxor Biotech, Paris, France). Total RNA (1 μg) was reverse-transcribed into first-strand cDNA using a High-Capacity cDNA Achieve Kit (Applied Biosystems, Foster City, CA, USA), according to the manufacturer’s instructions. RT-qPCR was performed using the fluorescent dye SYBR Green method, with SYBR Green PCR Master Mix in 384-well plates and the StepOnePlus system (Applied Biosystems). Amplification curves were analyzed according to the comparative cycle threshold method, using StepOnePlus software (version 2.1, Applied Biosystems by Life Technologies, Paisley, UK). The steady-state mRNA levels for the genes of interest were normalized against those of *GAPDH*.

### Cell uptake assay

For the microsphere uptake assay were performed as previously described^38^. The culture medium was renewed with medium containing fluorescent microspheres (yellow-green carboxylate-modified FluoSpheres, 100 nm, (Molecular Probes, Eugene, OR, USA)) and incubated overnight. After incubation, culture media were discarded and the cell monolayers were washed once with PBS before observation under a fluorescence microscope and under a confocal microscope (DMI 6,000 CS, Leica, Nussloch, Germany). The images were acquired with Leica LAS AF ((software version 3.3), available from https://leica-las-af-lite.software.informer.com/3.3) and analyzed with Image J software (version 1.51w, NIH, Bethesda, MD, USA).

For cytometry analyses, cells were detached from the plate with cold PBS, and fixed for 10 min in 4% paraformaldehyde in PBS. After the paraformaldehyde had been discarded, the MDMs were resuspended in PBS and analyzed by flow cytometry using a BD LSRFortessa X‐20 cell analyzer (BD Biosciences, San Jose, CA, USA). Dot plots of forward scatter (x axis) and side scatter (y axis) were used to gate viable cells prior to the detection of fluorescence emitted by the microspheres (on channel FL1-H) present inside the cells. The cytometry data were analyzed using FlowLogic software ((version 7.2.1, Inivai Technologies, Mentone, Australia), available from https://www.inivai.com/download/flowlogic). Results were expressed as the mean fluorescence intensity.

### Flow cytometry analysis of PBMCs

Blood samples from patients with COPD and from healthy blood donors were collected and incubated with 5 μL of anti-CD14-FITC, anti-CD16-PE and DRpc5-HLDR (Beckman Coulter, USA) antibodies for 10 min in the dark. Next, 2 mL of red cell lysis buffer (BD Biosciences, San Diego, CA, USA) were added for 10 min, and the tubes were centrifuged for 5 min at 500*g* and 20 °C. The supernatant was discarded, and the cell pellet was resuspended in 1× PBS. After further centrifugation for 5 min, the cell pellet was resuspended in 500 µL of 1% formalin in PBS. Measurements were performed with a FACSCalibur flow cytometer, and 200,000 events were obtained. T lymphocytes were excluded due to their lack of CD14, and natural killer cells and neutrophils were excluded due to their lack of HLDRA. The data were analyzed using Kalunza software (version 5, BD Biosciences). Results were expressed as individual values (%). Compensation was performed using antibodies with single-color fluorochromes for PE and FITC.

### Statistical analysis

The results were expressed as the mean ± standard error of the mean (SEM). The data were assessed in a one-way analysis of variance (ANOVA), followed by Bonferroni’s post-test. The threshold for statistical significance was set to p < 0.05. All statistical analyses were performed with GraphPad Prism software (version 6.0, GraphPad, San Diego, CA, USA).

## Data Availability

The datasets generated during and/or analysed during the current study are available from the corresponding author on reasonable request.
